# The genetics of phenotypic plasticity. XVII. Response to climate change

**DOI:** 10.1111/eva.12876

**Published:** 2019-10-31

**Authors:** Samuel M. Scheiner, Michael Barfield, Robert D. Holt

**Affiliations:** ^1^ Division of Environmental Biology National Science Foundation Alexandria VA USA; ^2^ Department of Biology University of Florida Gainesville FL USA

**Keywords:** climate change, evolutionary rescue, genetic assimilation, model, phenotypic plasticity

## Abstract

The world is changing at a rapid rate, threatening extinction for a large part of the world's biota. One potential response to those altered conditions is to evolve so as to be able to persist in place. Such evolution includes not just traits themselves, but also the phenotypic plasticity of those traits. We used individual‐based simulations to explore the potential of an evolving phenotypic plasticity to increase the probability of persistence in the response to either a step change or continual, directional change in the environment accompanied by within‐generation random environmental fluctuations. Populations could evolve by altering both their nonplastic and plastic genetic components. We found that phenotypic plasticity enhanced survival and adaptation if that plasticity was not costly. If plasticity was costly, for it to be beneficial the phenotypic magnitude of plasticity had to be great enough in the initial generations to overcome those costs. These results were not sensitive to either the magnitude of the within‐generation correlation between the environment of development and the environment of selection or the magnitude of the environmental fluctuations, except for very small phenotypic magnitudes of plasticity. So, phenotypic plasticity has the potential to enhance survival; however, more data are needed on the ubiquity of trait plasticity, the extent of costs of plasticity, and the rate of mutational input of genetic variation for plasticity.

## 1 INTRODUCTION

The world is changing at a rapid rate, threatening extinction for a large part of the world's biota. For some of those threats—over‐exploitation of resources, exotic toxins, intense nuclear radiation, the complete destruction of habitat—there is little that species can do. But for some types of threats, especially climate change, there are some potential responses that permit persistence (Holt, 1990). Climate change has occurred throughout the history of life on earth, and many species in their evolutionary history have experienced—and survived—such changes. Species survival can reflect two distinct modalities of coping with change. Climate change alters local conditions and possibly shifts optimal habitats to other locations. Given sufficient dispersal capacity, one potential response is a shift in a species’ range, although that becomes increasingly difficult as humans continue to fragment the landscape. Another potential response is to evolve to be able to persist in the altered conditions in the original habitat. The second response is the focus of this paper.

The effectiveness of both responses depends on the rate and magnitude of environmental change. Even with ample genetic variation, the rate of an evolutionary response can be outpaced by sufficiently rapid shifts in the environment. A potential solution is phenotypic plasticity (Burggren, 2018; Chevin, Collins, & Lefèvre, 2013). If traits are plastic, and if that plasticity is in a direction that maintains fitness, phenotypic change and fitness can potentially keep pace with environmental change. Even if that plastic change only partially moves trait values toward the fitness optimum, that may suffice to forestall or prevent extinction. Phenotypic plasticity has been and continues to be touted as a solution to the climate change crisis (Carter, Lynch, Myers, Rubenstein, & Thompson, 2019; Huey, Buckley, & Du, 2018; Matesanz, Gianoli, & Valladares, 2010; Merilä & Hendry, 2014; Nicotra et al., 2010; Valladares et al., 2014). In this paper, we contribute to a growing body of literature that assesses that assertion.

Through the use of an individual‐based simulation model, we examined the potential for phenotypic plasticity to mitigate species extinction in the face of climate change. In our model, trait values are not just phenotypically plastic, but plasticity itself has the potential to evolve. The current paper extends our previous effort (Scheiner, Barfield, & Holt, 2017) by making the pattern of environmental change more realistic and by making the initial plasticity develop as a result of selection on plasticity in the original environment. In our previous paper, we considered two scenarios: a single step change in the environment from one constant environment to another and a simple continual, directional change in the environment. We showed that phenotypic plasticity could increase the probability of population persistence in the absence of costs or limitations to plasticity. For both scenarios, there were no stochastic fluctuations to the environmental change, which are now included here. Such additional environmental variation can change the probability of persistence in two ways. First, increased variation can decrease the probability of persistence by continually changing the optimal phenotype and preventing the population from obtaining high fitness. Second, greater variation can increase the probability of persistence by selecting for greater initial phenotypic plasticity, allowing the population to better track the optimal phenotype. Which outcome is likely to prevail is unclear, depending on the interplay between the amount of deterministic and stochastic environmental variation and the cost of plasticity.

Our model confirms and extends analytic models that have considered similar patterns of environmental variation. Using a model with nonevolving plasticity, Chevin, Lande, and Mace (2010) showed that the existence of phenotypic plasticity could increase population survival in the face of continual, directional environmental change. In contrast, Nunney (2015) also used a model of nonevolving plasticity and found that plasticity decreased survival. This discrepancy, and comparisons with our results, is addressed in the Discussion. Using a model of a step change in the environment with evolving plasticity, Chevin and Lande (2010) showed that plasticity increased the probability of population survival, unless the benefits of plasticity were outweighed by high costs. Finally, modeling both a step change and continual change, but without plasticity, Chevin, Cotto, and Ashander (2017) showed that random environmental fluctuations can substantially increase extinction risk. Our simulations combine all of those elements—both a step change and continual directional environmental change, environmental fluctuations, and evolving plasticity—into a single model. The step and continual environmental change scenarios are meant to bracket the range of potential types of environmental variation resulting from climate change. The use of an individual‐based simulation allows more realism than previous analytic models, for example, by allowing genetic distributions to evolve in shape and variance as a result of selection, drift, and mutation, rather than just changing in mean value in response to selection (with constant shape and variance). Together, our results complement and advance those previous efforts.

We modeled a variety of scenarios. Because the heritability of plasticity tends to be lower than the heritability of mean trait values (Scheiner, 1993), we varied the magnitude of the plastic response of the phenotype (*b* in the model described below). We varied the within‐generation correlation of the environment at the time of development and selection (*ρ*), as that determines the optimal amount of plasticity (Gavrilets & Scheiner, 1993) that evolves in the ancestral environment. Because the pattern of climate change is uncertain, we varied the magnitude of the sudden change, the rate of the continual change, and the magnitude of the environmental variance (*τ*) added to both. Finally, because costs of plasticity can substantially constrain plasticity evolution (Botero, Weissing, Wright, & Rubenstein, 2015; DeWitt 1998; DeWitt, Sih, & Wilson, 1998; Fischer, Doorn, Dieckmann, & Taborsky, 2014; Lande, 2014; Sultan & Spencer, 2002), we compared evolution with and without costs. This extensive combination of scenarios was designed as broadly as possible to address our central question of the potential role of plasticity in mitigating species extinctions following environmental change.

## 2 MODEL STRUCTURE

We used an individual‐based model (implemented in Fortran 90) to simulate the effects of phenotypic plasticity on environmental rescue of a population in response to a change in the environmental mean value that happens either once (a step change) or continuously (a linear change). Random variation was added to this changing mean. The model is based on that of Bürger and Lynch (1995), to which we have added genetically determined phenotypic plasticity. In our model, individuals are diploid and hermaphroditic, with nonoverlapping generations. In each generation, juveniles are born at the same time, and each has a trait (phenotype *T*) that determines its survival to adulthood (when selection takes place). Random mating (including the possibility of selfing) and reproduction then occur, after which all adults die.

### 2.1 Determining the phenotype

An organism's total phenotype is the sum of three components: contributions from nonplastic loci, contributions from plastic loci whose effect depends on the developmental environment, and a random component. The phenotype (*T_ij_*) of the *i*th individual developing in generation *j* is given by:

(1) $$T_{\mathrm{ij}}=\sum_{k=1,2n}N_{\mathrm{ijk}}+E_{j}b\sum_{k=1,2m}P_{\mathrm{ijk}}+z_{\mathrm{ij}},$$

where *N_ijk_* are allelic values at the *n* (10) nonplastic loci and *z_ij_* is a zero‐mean, unit‐variance‐independent Gaussian random deviate (the random component of phenotype); these are the components of the original model of Bürger and Lynch (1995). (See Table 1 for a list of all variables and parameters.) The middle term is the contribution of plasticity to the phenotype, where *P_ijk_* are allelic values at the *m* (10) plastic loci of this individual and *E_j_* is the environment at the time of trait development in generation *j*. For a clone of genetically identical individuals, taking the expected value of Equation (1) gives *E*[*T_ij_*] =*a* + *dE_j_*, which is a linear reaction norm (mean phenotype is a linear function of the environment) with an intercept of *a* (the sum of nonplastic alleles) and a slope (*d*) equal to the product of parameter *b* and the sum of plastic allelic values. The plasticity parameter (*b*) determines the magnitude of the plastic response by the phenotype for a given plastic genetic value, scaling the phenotypic variation due to plasticity relative to the variation due to the nonplastic alleles. For a value of *b* = 1, the sum of alleles at plastic loci would be 1 for individuals perfectly adapted by plasticity alone (for perfect adaptation without plasticity, the sum of the nonplastic alleles would be *E_j_*).

**Table 1:** Variables and parameters

Symbol	Meaning	Value
Variables		
*T*	Phenotype of the individual	
*N*	Nonplastic allelic value	
*P*	Plastic allelic value	
*E*	Environment at the time of development	
*z*	Random component of the phenotype	
*T_opt_*	Optimum phenotype at the time of selection	
*W*	Individual survival probability from juvenile to adult	
${\bar{T}}_{\mathrm{opt}}$	Expected value of the optimum phenotype at selection	
*i*	Subscript for *i*th individual	
*j*	Subscript for *j*th generation	
*k*	Subscript for *k*th allele	
Parameters		
*b*	Plasticity parameter	0.0 – 1.0
*ρ*	Correlation between the environments at time of development and the time of selection	0.25, 0.50, 0.75
*τ*	Environmental standard deviation	0.25, 0.50, 0.75, 1.0
*n*	Number of nonplastic loci	10
*m*	Number of plastic loci	10
*ω*	Strength of selection	1
*c*	Indicator variable for cost of plasticity	0 or 1
*ω_P_*	Cost of plasticity	1
*μ*	Per‐locus mutation rate	0.0005
*α* ^2^	Variance of mutation effect	0.05

### 2.2 Selection and plasticity costs

Selection occurred during survival from juvenile to adult, which had a probability (*W_ij_* for the *i*th individual in generation *j*) that was a Gaussian function of the difference between the individual's phenotype and an optimum phenotype for its environment (*T*_opt_ *_,j_*):

(2) $$W_{\mathrm{ij}}=\exp\left\{-\frac{1}{2}{\left(\frac{T_{\mathrm{ij}}-T_{\text{opt},j}}{\omega }\right)}^{2}\right\},$$

where *ω* determined the strength of selection on the phenotype (a lower value being stronger selection). Fitness is the product of *W_ij_* and fecundity. The survival probability was 1 for an individual with trait *T_ij_* equal to the optimum *T*_opt_ *_,j_* and decreased as the absolute value of the difference between *T_ij_* and *T*_opt_ *_,j_* increased. The effective selection was stabilizing when the population mean trait value was near the optimum, and directional when the mean was far from the optimum.

To model a cost of plasticity, we modified the survival function by allowing stabilizing selection on the sum of the plasticity alleles around 0 (i.e., any departure from 0 lowers fitness) as in Chevin and Lande (2010). Including a cost of plasticity, the survival probability became:

(3) $$W_{\mathrm{ij}}=\exp\left\{-\frac{1}{2}{\left(\frac{T_{\mathrm{ij}}-T_{\text{opt},j}}{\omega }\right)}^{2}-\frac{c}{2}{\left(\frac{\sum_{k}P_{\mathrm{ijk}}}{\omega_{P}}\right)}^{2}\right\},$$

where *c* is 1 if there is a cost of plasticity and 0 if not, and *ω_P_* determines (inversely) the cost of plasticity (*ω* = *ω_P_* = 1 for all simulations). This cost of plasticity was independent of allelic expression; even when plasticity was not expressed (i.e., *E_j_* = 0), the plasticity loci still had an effect on fitness. An example of such an independent cost would be maintaining additional cellular or organismal machinery needed to translate an environmental signal into a phenotypic change (DeWitt et al., 1998). Alternative forms of cost could be either a fixed cost, given the existence of any nonzero plastic alleles, or a cost that scales with the phenotypic expression of the plastic alleles, not just their genotypic values. In our previous paper (Scheiner et al., 2017), we concluded that these alternatives might have changed the details of the evolutionary dynamics, but not the general trends.

### 2.3 Reproduction and mutation

Reproduction followed selection. Density was regulated by limiting the number of matings to a value of 256. If there were fewer than 256 adults, then all adults mated as a female; if there were more than 256, that number was chosen at random (without replacement) to act as females. (This mating system differs from that in Bürger and Lynch (1995), which included a weak Allee effect.) Each mating female was paired with an adult randomly selected with replacement to act as a male; this could be the same individual as the female (i.e., selfing was allowed). Each mated pair produced 4 offspring. Because mating was random, the degree of selfing depended only on population size and was only significant when the population size was small, which occurred only for some of the step change simulations and only for a brief period. Previous papers have varied population size or fecundity and found that they did not affect qualitative patterns (Barfield & Holt, 2016; Holt & Gomulkiewicz, 2004; Orive, Holt, & Barfield, 2018; Scheiner et al., 2017).

During reproduction, there was free recombination. Allelic values could take on any real value. Each offspring haplotype mutated with probability (*n* + *m*)*µ*, *µ* being the per‐locus mutation rate (*n* = *m* = 10 and *μ* = 0.0005 for all simulations). If a mutation occurred, a random locus was selected and a zero‐mean Gaussian with variance *α* ^2^ (*α* ^2^ = 0.05 for all simulations) was added to the previous allelic value; the mutation rate and variance for plastic and nonplastic alleles were the same. After the alleles of each offspring were determined, the random component of its phenotype was chosen. Because the plasticity parameter (*b*) determines how genetic variation gets translated into phenotypic variation, a greater value means that the same mutational change has a greater phenotypic effect. A population with a higher plasticity parameter therefore would be expected to be able to plastically generate new phenotypic variation at a higher rate.

### 2.4 Initial conditions

For all simulations, a population was initiated with 256 adults with random nonplastic and plastic alleles. There was then a 2000‐generation equilibration period, with the mean phenotypic optimum held constant at 0, to allow the population to reach mutation–selection–drift equilibrium. For generation *l*, the environment at the time of development (*E_l_*) was determined by drawing an independent zero‐mean Gaussian with variance *τ* ^2^. The optimum at the time of selection for that generation (*T_opt,l_*) was another zero‐mean Gaussian with variance *τ* ^2^ and a correlation coefficient of *ρ* with *E_l_*, calculated as $T_{\text{opt},l}=\rho E_{l}+\tau \sqrt{1-\rho^{2}}\xi_{l},$ where *ξ_l_* is an independent zero‐mean, unit‐variance Gaussian deviate. This variation in the environment determined the amount of plasticity at the end of the equilibration period. We always set the cost of plasticity equal to 0 during the equilibration period, so the mean plasticity slope was expected to equilibrate at a value of *ρ*. The initial value of each allele was an independent Gaussian. We performed initial simulations over a range of parameter sets to determine the means and variances that would speed the approach to equilibrium within the 2000 generations. Initially, nonplastic alleles had a mean of 0 and a variance of 0.02/(2*n*), while plastic alleles had a mean of *ρ/*(2*m*) and a variance of the lower of either 0.375/(2*m*) or 0.05/(2*mbτ*). These initial values allowed the population genetics to equilibrate within the 2000‐generation period.

### 2.5 Environmental change

After the equilibration period, for the step change the mean environment was abruptly increased to a fixed positive value; for continual change, the mean environment increased by a fixed increment each generation, resulting in a linear change over time. In either case, the value of the environments at the time of development and at the time of selection in generation *j* was determined by taking the mean environment and adding a random value. The procedure was similar to that used during the equilibration period. The environment at the time of development was the sum of the mean environment and an independent zero‐mean Gaussian with variance *τ* ^2^ (*e_j_*), while the environment at the time of selection was the sum of the mean environment and $\rho e_{j}+\tau \sqrt{1-\rho^{2}}\xi_{j},$ where *ξ_j_* is an independent zero‐mean, unit‐variance Gaussian deviate. In some cases, the variance of the random environmental component differed between the equilibration period and the environmental change period. (Throughout the manuscript, “initial” refers to the time the environment changed or started to change following the equilibration period, and all time plots start then.) The magnitude of the step change varied from 2 to 6, while for continual change, the total change varied from 20 to 200, depending on the rate of change; for comparison, the width of the fitness function, *ω*, was 1. The difference between the environments at the time of selection and development was Gaussian with a mean of 0 and a variance of 2(1 – *ρ*)*τ* ^2^, so the expected value of the magnitude of the difference was $2\tau \sqrt{\left(1-\rho \right)/\pi }.$ With our standard parameters, this is about 0.4, while its maximum value is less than 0.8, somewhat less than the step change but much less than the total continual change. Unless otherwise indicated, after the change occurred or started all simulations were continued for 1,000 generations or until the population went extinct. For continual change, in our model the phenotypic response of a plastic individual got larger, even if there were no additional genetic changes, because of the change in the environmental inducer. Thus, we assumed that there were no intrinsic morphological, physiological, or developmental limits to the plastic response.

### 2.6 Parameter sets and response variables

In addition to the probability of population persistence, we present results for relative phenotype and relative plasticity, which are values of mean phenotype and plasticity normalized to the mean optimum phenotype at the time of selection. The relative phenotype for a population is therefore its mean phenotype divided by the mean optimum phenotype $\left({\bar{T}}_{\text{opt},j}\right),$ so that a perfectly adapted population has a relative phenotype of 1. Its relative plasticity is the mean plastic component of phenotype [the middle term on the right side of Equation (1)] divided by ${\bar{T}}_{\text{opt},j},$ which is a measure of the contribution of the plastic alleles to adaptation (one minus the relative plasticity is the contribution due to the nonplastic alleles). A population with a relative plasticity of 1.0 can remain at a changing optimal phenotype without any evolution of the nonplastic alleles because the middle term of Equation (1) is always equal to *E_j_*, which has a mean of ${\bar{T}}_{opt,j}.$

We determined these quantities with and without a cost of plasticity for both types of environmental change. For each, we varied the plasticity parameter *b*, the degree of change of the environment, and the within‐generation correlation in environmental variation. To examine the cost of plasticity, we set *c* to 0 or 1. When both *b* = 0 and *c* = 0, our model matches previous models without plasticity and serves as a baseline for comparison. For parameter values of *b* = 0 and *c* = 1, the plasticity loci incur a fitness cost but never affect trait values and serve as an indicator of just the cost effect.

For each parameter set, 1,000 replicates were run and the probability of persistence was the fraction of such populations that were not extinct after a specified period (1,000 generations). For each parameter set, at each generation we calculated the mean phenotype (*T_ij_*) of the population, its mean expressed plasticity (*E_j_b*Σ*P_ijk_*), the variance in phenotypes, the genetic variances of the plastic (Σ*P_ijk_*) and nonplastic components (Σ*N_ijk_*) of the genotype, and the fraction of populations that survived to that generation. These variables (except the last) were averaged over all populations that survived to the end of the simulation. In the results, we show final values of these quantities and, in some cases, their time courses.

## 3 RESULTS

### 3.1 Step change—no costs

**Figure 1:**
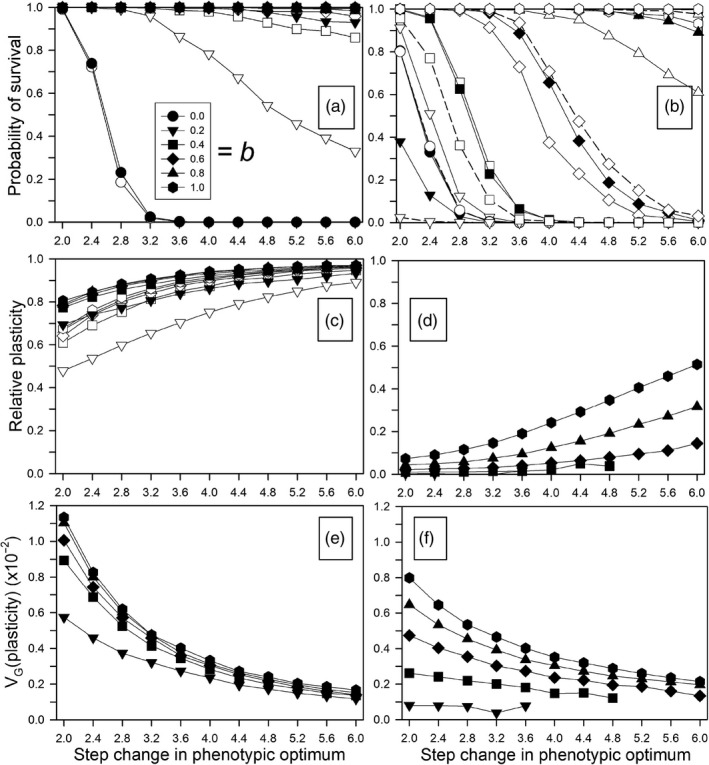
Responses after 1,000 generations to a step change in the environment for different plasticity parameters (*b*) and different magnitudes of the correlations between the environments at development and selection (*ρ*: 0.25 = open symbols, solid lines; 0.50 = solid symbols; 0.75 = open symbols, dashed lines). Other parameters were as follows: *τ* = 0.5. (a, c, e) Without plasticity costs. (b, d, f) With plasticity costs. (a, b) The probability of survival. (c, d) Final relative plasticity. (e, f) Final genetic variation for plasticity (variance of ∑*P_ijk_*). When only solid symbols are shown, the amount of temporal autocorrelation made little or no difference

The probability of survival was greatly enhanced by the presence of noncostly phenotypic plasticity (Figure 1a). A larger within‐generation environmental correlation (*ρ*) selected for greater initial phenotypic plasticity, and that greater plasticity resulted in higher survival for a given size of the step change. The increased plasticity due to the greater environmental correlation at the time of the step change persisted to the end of the simulations (Figure 1c). However, these effects of initial plasticity were seen only for small values of *b*. Otherwise, for all correlations the final amount of plasticity was very high and survival probabilities at or near 1.0. The presence of environmental fluctuations greatly enhanced selection for plasticity and the probability of survival (compare Figs. 1A and 1C with Figs. 1a and 5a of Scheiner et al., 2017). The amount of genetic variation for plasticity at generation 1,000 declined as the size of the step change increased (Figure 1e).

**Figure 2:**
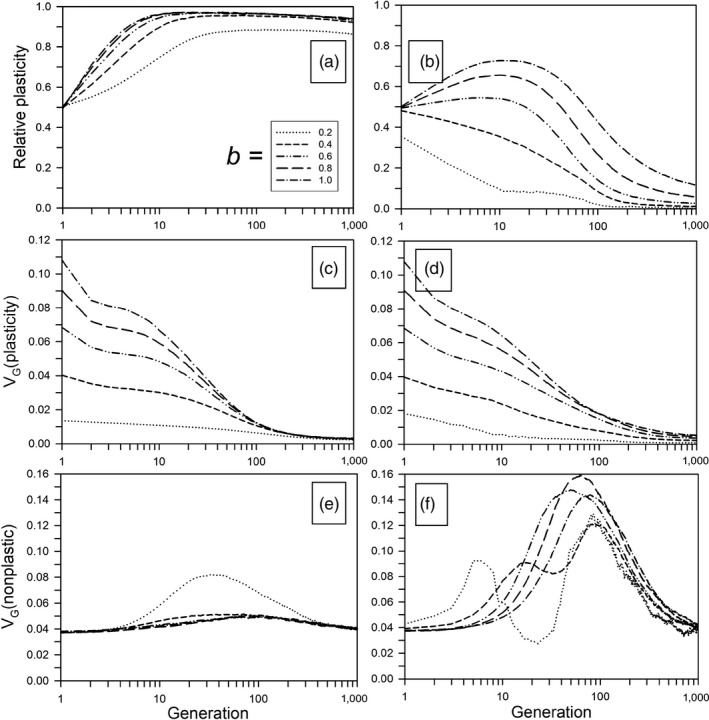
Temporal dynamics following a step change in the environment for different plasticity parameters (*b*). Other parameters were as follows: *ρ* = 0.5, *τ* = 0.5. (a, c, e) Without plasticity costs and with a step change in the environment of 4.0 units. (b, d, f) With plasticity costs and a step change in the environment of 2.8 units. (a, b) Relative plasticity. (c, d) Genetic variation for plasticity (∑*P_ijk_*). (e, f) Genetic variation for nonplastic phenotype component (∑*N_ijk_*)

Immediately after the step change, phenotypic plasticity increased quite quickly, often reaching close to its maximal value in about 10 generations (Figure 2a). Following this increase, selection gradually returned plasticity to the level set by the within‐generation environmental correlation, but that process took up to 100,000 generations (Figure S1). This very slow decline in phenotypic plasticity occurred because the amount of genetic variation for plasticity was quickly eroded (Figure 2c). In contrast to the plastic genetic component, the amount of genetic variation for the nonplastic component stayed relatively constant, except for a transient rise at the lowest value for *b* (Figure 2e). In this case, the plasticity variance was also quite low (Figure 2c), so the rise in plasticity was slower and peaked later and at a lower level than with higher values of *b* (Figure 2a). Therefore, there was greater selection on nonplastic alleles.

Besides the magnitude of the correlation (*ρ*), the magnitude of the environmental fluctuations (standard deviation *τ*) also affected the probability of survival and selection on phenotypic plasticity. The magnitude of the fluctuations prior to the step change determines the variation in the amount of plasticity at the end of the equilibration period, with higher fluctuations resulting in less variation around the expected value for a given magnitude (*ρ*). After the step change, higher fluctuations resulted in a greater difference (on average) between the environments of development and selection, and a faster return of plasticity toward the expected value as determined by *ρ*. In order to control for the effects of selection on plasticity prior to the step change, we considered two scenarios: the same magnitude of fluctuations before and after the change (Figure S2) and a single, intermediate magnitude before the change with different magnitudes after (Figure S3). The magnitude of the fluctuations had a small effect on the probability of survival, which was high in all cases (Figures S2A, S3A), with almost no effect on the amount of genetic variation for plasticity (Figures S2E, S3E). Greater fluctuations resulted in lower amounts of phenotypic plasticity after 1,000 generations, mostly because of stronger selection on plasticity to return it to the prechange level (Figures S2C, S3C).

Although the probability of survival did not vary much, for the simulations for which *τ* only differed after the environmental change (Figure S3A), the probability of survival was consistently lower with higher fluctuations, presumably because of the greater average difference between the environments of development and selection. When *τ* was kept constant for each run (Figure S2A), the probability of survival was lower for the lowest and the highest values of *τ*. A likely explanation for the lower survival for the lowest value of *τ* is that with low *τ*, the population average plasticity varied more around *ρ* during the equilibration period so that some populations had a low average plasticity at the moment when the environmental change occurred and subsequently failed to persist.

### 3.2 Step change—costs

The imposition of a cost of plasticity substantially decreased the probability of survival (Figure 1b). As with our previous model without environmental fluctuations, the effects of plasticity were complex (compare with Fig. 1b of Scheiner et al., 2017). A small plasticity effect—a low value of *b*—decreased the probability of survival relative to having no plasticity because the cost of plasticity (which depends only on the magnitude of the plasticity allelic values, not the realized phenotype) decreased fitness without providing a sufficient shift in the phenotype to compensate during the critical early generations. Similarly, the higher within‐generation correlation, which selects for greater plasticity, can reduce the probability of survival [for *b* = 0.4 (square symbol) compare the dashed line (*ρ* = 0.75) with the solid lines (*ρ* = 0.25, 0.50)]. These effects reversed for greater values of *b*, in which the plasticity benefit outweighed the cost. Not surprisingly, the final relative plasticity was lower than the no‐cost case and lower than when fluctuations were absent (compare Fig. 1D with Fig. 1b of Scheiner et al., 2017). On the other hand, the amount of plastic genetic variation at 1,000 generations was greater than either the no‐cost or no‐fluctuation scenarios, likely because of the decreased selection on plasticity (compare Fig. 1F with Fig. 5b of Scheiner et al., 2017).

As with the no‐cost scenario, the amount of plasticity reached its maximum between 10 and 100 generations, after which it decreased much more rapidly than without a cost, except for the lowest values of *b* where it was always decreasing (Figure 2b). Unlike the no‐cost scenario, the amount of genetic variation for the nonplastic component rose significantly for all *b* values, before gradually returning to its initial value by 1,000 generations (Figure 2d). Plasticity substantially increased the initial population persistence, but because it was costly, adaptation switched to evolution of the nonplastic alleles and plasticity waned. This nonplastic adaptation resulted in a temporary increase in the nonplastic genetic variance (Figure 2f).

The magnitude of the environmental fluctuations (*τ*) had much larger effect on the probability of survival, compared to the no‐cost scenario, especially when the magnitude was the same before and after the step change (Figures S2B, S3B). The greater the amount of the fluctuations the lower the probability of survival, because again it resulted in more plasticity and a decrease in fitness due to those costs in the critical early generations. The magnitude of the fluctuations had less effect on the amount of plasticity at generation 1,000 (Figures S2D, S3D) than in the no‐cost scenario, because the plasticity reached low levels by this time due to its cost. The amount of genetic variation for plasticity was not affected by the magnitude of the fluctuations (Figures S2F, S3F), as with the no‐cost scenario, although increasing the magnitude of the step change decreased plasticity variance less with a cost than without a cost. The value of *b* used for the cost scenario (0.6) was greater than the no‐cost scenario (0.2), because those values were in a range to see the effects. Lower values of *b* with a cost would have resulted in almost no survival, and higher values for the no‐cost scenario would have resulted in no differences in the amount of plasticity (and 100% survival).

### 3.3 Continual change—no cost

**Figure 3:**
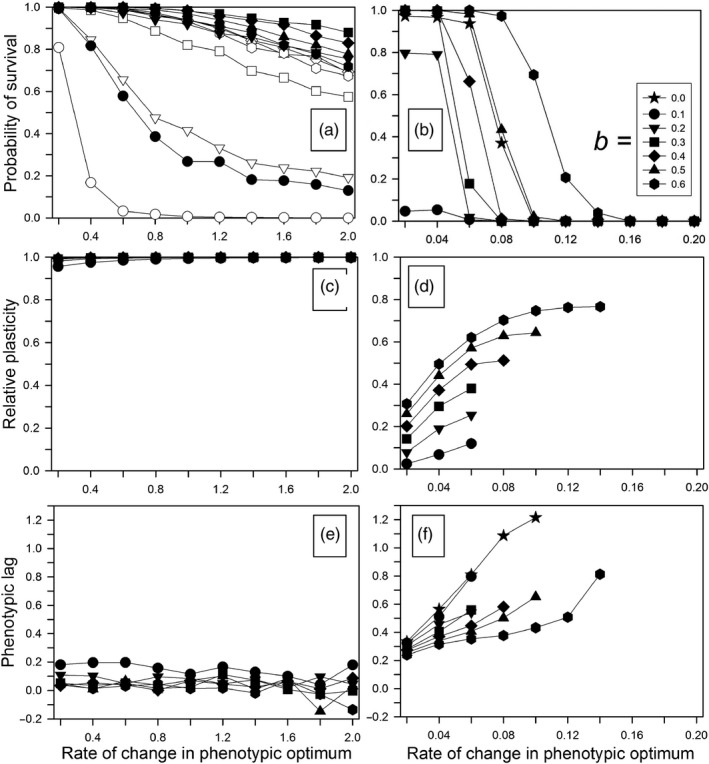
Responses after 1,000 generations of continual environmental change for different plasticity parameters (*b*) and different magnitudes of the correlations between the environments at development and selection (*ρ*: 0.25 = open symbols; 0.50 = solid symbols). Other parameters were as follows: *τ* = 0.5. (a, c, e) Without plasticity costs. (b, d, f) With plasticity costs. (a, b) The probability of survival. (c, d) Average final relative plasticity. (e, f) Expected value of final phenotypic lag (average optimum phenotype minus average phenotype). When only solid symbols are shown, there was little or no difference as a function of the amount of temporal autocorrelation

**Figure 4:**
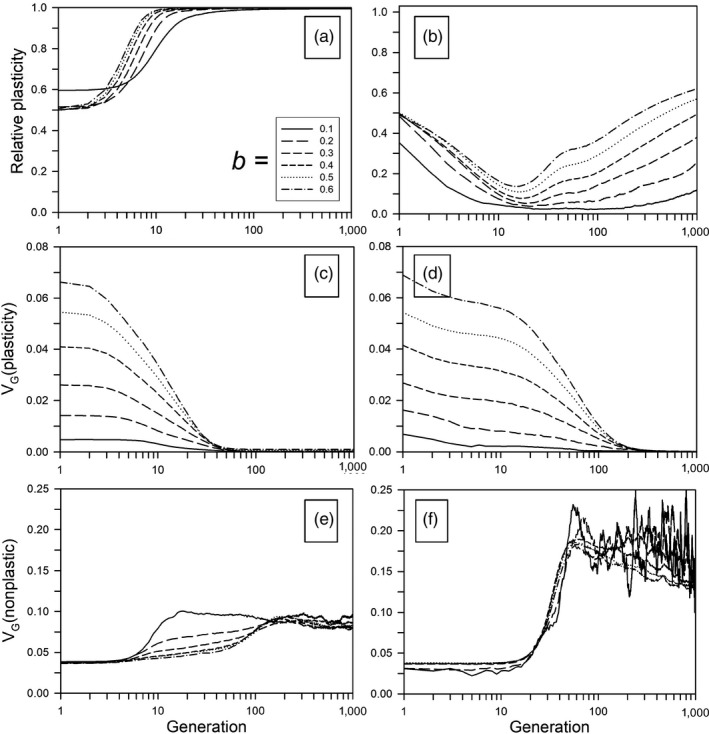
Temporal dynamics during continual environmental change for different plasticity parameters (*b*). Other parameters were as follows: *ρ* = 0.5, *τ* = 0.5. (a, c, e) Without plasticity costs and a rate of environmental change of 1.2 units/generation. (b, d, f) With plasticity costs and a rate of environmental change of 0.06 units/generation. (a, b) Average relative plasticity. (c, d) Average genetic variation for plasticity (∑*P_ijk_*). (e, f) Average genetic variance of nonplastic alleles (∑*N_ijk_*). All values were averaged over all populations that survived until the end of the simulation. For *b* = 0.1 without a cost, populations that survived had a higher than average initial plasticity, while with a cost, surviving populations had a lower than average initial plasticity. Therefore, in A and B the *b* = 0.1 curves do not begin at *ρ* = 0.5

When the environmental change included a continual, directional component, and when plasticity was not costly, greater amounts of plasticity (higher *b* or higher *ρ*) substantially increased the probability of survival for the first 1,000 generations (Figure 3a). The no‐plasticity scenario (*b* = 0) is not shown because it resulted in zero survival over the entire range of rate changes used here (from Scheiner et al., 2017, Fig. 9a, with *b* = 0 all populations survived for a change of 0.05 per generation and none for a change of 0.15). Extinction, when it occurred, happened almost exclusively in the first 20 to 40 generations (Figure S4A); populations quickly either went extinct or evolved high plasticity, which allowed them to follow the changing optimum indefinitely. The relative plasticity evolved to 1.0 within 100 generations (Figure 4a), at which point the genetic variation of plasticity was very low (Figure 4c). Over the first 200 generations, the amount of genetic variation of the nonplastic component rose somewhat (Figure 4e). Overall, the probability of survival and the final relative plasticity were greater than for the previous model that did not include environmental fluctuations (Scheiner et al., 2017). For our previous model, the initial relative plasticity was 0 or 0.2, compared to 0.5 here, so plasticity did not evolve to as high a value and, therefore, populations went extinct at lower rates of change.

Phenotypic lag measures the distance of the population mean phenotype from the optimal phenotype. (For the latter, we used the expected value of the optimal phenotype, not including the random component.) That lag was close to zero for all rates of change, and little affected by the amount of plasticity, commensurate with a relative plasticity of 1.0 (Figure 3e). This lag was a little lower than for the model without environmental fluctuations (compare with Fig. 9e of Scheiner et al., 2017).

A greater magnitude of environmental fluctuation after the start of the environmental change (with the same magnitude before the change) decreased the survival probability (Figure S6A); as with the step change, this is likely because of the increased average difference between the environments of development and selection, increasing the amount of maladaptation. Also as with the step change, the pattern with a constant *τ* is more complex, with only the highest *τ* showing clearly lower survival, likely because of contrasting effects of the magnitude of variation before and after the change. In both cases, *τ* had almost no effect on either the amount of plasticity, which was always close to 1.0 (Figures S5C, S6C), or the phenotypic lag (Figures S5E, S6E).

### 3.4 Continual change—cost

When plasticity was costly, it substantially decreased the probability of survival (Figure 3b; note the difference in scale for the rate of change between the left panels of Figure 3, with no cost of plasticity, and the right panels, with a cost). This survival probability was lower than when plasticity was absent (*b* = 0), except for the greatest amount of plasticity. This result is very similar to the model that lacked environmental fluctuations (see Fig. 9b of Scheiner et al., 2017). The amount of plasticity decreased for the first 10–20 generations (except for *b* = 0.1) before rising steadily again, giving a final plasticity approximately proportional to the value of *b*, again similar to the no‐fluctuation model (compare Figs. 3D and 4D with Figs. 9d and 11b of Scheiner et al., 2017). (Our previous model started with no initial mean plasticity and so does not show the early decline in plasticity.)

Extinction in some cases occurred soon after the change began. If it did not, there was a period with few extinctions, after which survival decreased steadily over time (Figure S4b). The initial extinctions were almost certainly due to the cost of plasticity, which was especially high for low *b* because the cost was based on the sum of the plasticity alleles and that sum had to be greater with lower values of *b* to achieve the same phenotypic effect. In the first 10–20 generations, the mean phenotypic optimum did not change enough to cause significant maladaptation and the plastic change to the phenotype was small, so that the cost of plasticity caused the amount of plasticity to decrease. Extinctions were rare during the low‐plasticity period, because the plasticity cost was low and the population phenotype could still keep up with the changing optimum (note the increase in the nonplastic variance in Figure 4f). In fact, the nonplastic alleles alone would have allowed a high persistence with no plasticity at this rate of change; the survival at *b* = 0 was over 90% for a rate of change of 0.06 (Figure 3b). Without the cost of plasticity, survival would have been near 100% (Orive et al., 2018). After the initial decrease, plasticity increased steadily and eventually became high enough that its cost caused populations to start going extinct. So, higher plasticity was apparently selected for even though it caused population extinctions that would not have occurred at low (or zero) plasticity. Our previous model (Scheiner et al., 2017) without fluctuations in the optimum phenotype showed the same pattern. Thus, the evolution of plasticity may not always facilitate persistence in an altered environment, but indeed can even hamper such persistence.

The phenotypic lag was greater when costs were present, but no different than in the absence of environmental fluctuations (compare Fig. 3F with Figs. 9f of Scheiner et al., 2017). The amount of genetic variation for plasticity declined over the first 1,000 generations (Figure 4d), albeit more slowly than in the absence of plasticity costs. Coincident with the decline in the genetic variation for plasticity was a substantial rise in the genetic variation for the nonplastic component (Figure 4f). A greater magnitude of the environmental fluctuation (*τ*) decreased the probability of survival (Figures S5B, S6B), but had no effect on the amount of plasticity (Figures S5D, S6D) or the phenotypic lag (Figures S5F, S6F).

## 4 DISCUSSION

### 4.1 Good news, bad news

The potential for phenotypic plasticity to enhance survival and adaptation in the face of climate change is a mix of good news and bad news. Our model provides a best‐case scenario for the role of phenotypic plasticity, and our results should be considered in that light. First, the good news is that plasticity can at times enhance survival and adaptation; the bad news is that current evidence suggests that the majority of traits are not adaptively phenotypically plastic. Palacio‐López, Beckage, Scheiner, and Molofsky (2015), in a meta‐analysis of plant traits, found that about half the traits did not show phenotypic plasticity, and only about a third showed adaptive plasticity. Similarly, Acasuso‐Rivero, Murren Courtney, Schlichting Carl, and Steiner Ulrich (2019), using a different meta‐analytic approach and examining a variety of organisms, concluded that nonadaptive or maladaptive plasticity might be common. Second, the bad news is that costs of plasticity can severely reduce the potential for survival and adaptation. The good news is that there is currently little evidence for extensive costs of plasticity (Murren et al., 2015). Third, the good news is that under the parameters of our model, there was sufficient genetic variation for plasticity so that it was able to evolve quickly enough to respond to both the step and continual change. The bad news is that the heritability of trait plasticity is generally lower than within‐environment trait heritability (Scheiner, 1993). Fourth, the bad news is that developmental and evolutionary limits, which we did not include in our model, might limit the ability of plasticity to rescue real populations. The good news is that while climate change is likely to continue for a century or more, the equivalent of tens of generations for many organisms, it is unlikely that there will be continual directional change for the thousand generations of our model. The short‐term dynamics that we model may be within the developmental limits of many traits and organisms.

The differences in results among the models of Chevin et al. (2017), Nunney (2015), and those in this paper reflect differences in assumptions. First, all three models found that costs of plasticity restricted its potential to prevent extinction in the face of continual environmental change. For all models, greater amounts of plasticity were associated with greater costs. Depending on the details of the model, the threshold at which costs exceeded benefits of plasticity differed, leading to differences in the exact circumstances in which plasticity would be favored (compare Fig. 3B with Fig. 2 of Chevin et al., 2017 and Tables 1 and 2 of Nunney, 2015). The most significant difference between our model and the previous models is that we allowed plasticity to evolve, meaning that it could potentially reach the adaptive optimum. Nunney’s (2015) conclusion that plasticity was almost always detrimental was due to his assumption that plasticity was nonevolving and restricted to being maladaptive, with a reaction norm slope less than the optimal value. In his model, the greater the amount of evolution of the nonplastic genetic component of the phenotype, the greater the amount of maladaptation expressed by the plastic component.

### 4.2 Patterns of environmental fluctuations

We varied two components of the environmental fluctuations: the within‐generation correlation between the environment of development and the environment of selection, and the magnitude of those fluctuations. In general, neither component had a very large effect on evolutionary outcomes. The biggest effect was on survival in response to continual change in the absence of costs (Figure 3a). Because the magnitude of the correlation determined the magnitude of plasticity at the start of the environmental change, for small values of *b* higher correlations were associated with substantially higher rates of survival. Otherwise, effects were small and did not change the overall conclusions. The small effects of the magnitude of the fluctuations on persistence are encouraging as climate change is expected to increase overall climatic variability and the likelihood of extreme events. Not all climate change is directional. While global mean temperatures are rising, precipitation may increase, decrease, or simply become more variable depending on the region.

Our model did not include spatial variation. We modeled a single population with temporal variation, as have related models (Chevin et al., 2017, 2010; Chevin & Lande, 2010; Nunney, 2015). Yet, we know that spatial variation can interact with temporal variation to affect selection for or against phenotypic plasticity (Scheiner, 2013). Additionally, our model did not include an among‐generation correlation of the environment of development or the environment of selection, which again is known to affect selection on plasticity (Scheiner, 2013). Nor did the model include other related phenomena, such as the potential influence of parental phenotypes on offspring phenotypes (e.g., epigenetic inheritance or transgenerational plasticity, Herman & Sultan, 2011; Mousseau & Fox, 1998). Future models should explore these possibilities to see whether they alter our general conclusions.

### 4.3 Genetic assimilation

A previous analytic model (Lande, 2009) considered the evolution of plasticity in response to a step change in the environment combined with within‐generation variation before and after the change, similar to our model. Our conclusions are consistent with that of Lande in that in the absence of plasticity costs, the return time to the magnitude of plasticity set by the within‐environment correlation is a very long process (Figure S1). And if there is no within‐generation variation so that there is no selection on plasticity, the magnitude of plasticity does not decrease at all (Scheiner et al., 2017). Any decrease in the magnitude of plasticity requires either some sort of cost to plasticity, a limitation to plasticity, or direct selection on plasticity. Even then, it is a slow process, which in our simulations was much slower than in Lande (2009). This rate difference might be because he assumed that the genetic variance for plasticity is fixed, whereas in our model genetic variation had to be generated by mutation and be depleted by selection and drift, an often a slow process. Thus, the process of genetic assimilation—the replacement of plasticity by fixed trait differences—is likely to be rare given the likelihood of additional changes in the environment that would once again favor increased phenotypic plasticity. These results point to an additional explanation for hyperplasticity—plasticity that is greater than the optimum (Scheiner & Holt, 2012); hyperplasticity may simply be the result of selection for greater plasticity in the past, rather than a current adaptation.

### 4.4 Conclusions

Our results broadly concur with prior studies that phenotypic plasticity (and its evolution) can often foster population persistence in temporally changing environments. In some circumstances, even though extinction ultimately occurs, plasticity can be selected for and act to delay that extinction (e.g., Figure 3b; Figure S4). Articulating the circumstances in which evolution can forestall extinction (e.g., through evolutionary rescue, Bell, 2013; Carlson, Cunningham, & Westley, 2014; Gomulkiewicz & Holt, 1995) is useful, but needs to be bracketed with an appreciation that evolutionary processes can at times tilt populations toward the cliff of extinction (i.e., evolutionary suicide, Parvinen, 2005; Webb, 2003). For example, our study revealed that at times the evolution of plasticity, when there were costs, could make it more difficult for a population to persist in a continually changing environment. Most prior theoretical examples of evolutionary suicide have involved frequency‐dependent selection; our examples do not.

Evolutionary responses to climate change and other long‐term environmental perturbations depend on the evolutionary dynamics of heritable variation itself. Many current approaches to eco‐evolutionary dynamics (e.g., adaptive dynamics, or quantitative genetics approaches) make simplifying assumptions about the genetic variation that permits evolution by natural selection. Our individual‐based simulations reveal that there can be strong, and at times surprising, patterns that emerge in or because of the dynamics of genetic variation. A species may show high plasticity over long spans of evolutionary history, not because of relentless selection for plasticity, but because a past bout of strong selection led to fixation of a plastic phenotype. At the same time, that selection may corrode available heritable variation in plasticity so that future reductions in plasticity are constrained. Moreover, the dynamics of genetic variation in the nonplastic and plastic components of a phenotype can be mutually interdependent, leading to complex temporal patterns in genetic variation (e.g., Figure 2f, dotted line). Observed patterns of plasticity, and realized levels of genetic variation in plasticity, may reflect not just current selection, but patterns of temporal variation in the environment stretching far into the past. These dynamics of heritable variation have been largely untouched in empirical analyses of phenotypic plasticity, but are critical to understanding the possible outcomes of climate change.

Our results point to a need for additional information about phenotypic plasticity coupled with more specific models. The meta‐analysis of the frequency of plasticity (Palacio‐López et al., 2015) was restricted to herbaceous plants. That analysis was aided by the existence of many reciprocal transplant studies of species with short lifespans. Information is likely limited for allowing more general analyses (e.g., for long‐lived species or mobile animals, Sgrò, Terblanche, & Hoffmann, 2016). Additional attempts to measure costs of plasticity are warranted, but those measures should focus on traits that are likely to be the target of shifting climates. How realistic are developmental limits on adaptive phenotypic plasticity, and do those same developmental limits hold regardless of whether the trait develops in a plastic or a nonplastic manner? Predictions about long‐term persistence need more information about developmental constraints. Finally, there is no information on the rate of mutational input to plasticity variation. Such studies need to be done in a way that measures not only mutation rates per se, but how that mutational variation gets translated into phenotypic variation (the *b* parameter in our model). Our model allowed us to explore a variety of general scenarios. Models and information on particular species will be necessary to predict and manage climate change responses.

## AUTHOR CONTRIBUTIONS

All authors contributed to the development of the ideas and the writing of the manuscript. The models were coded and run by MB.

## Data Availability

No data were generated in this study.
