# Supplementary material for: The genetics of phenotypic plasticity. XVII. Response to climate change
Source: Evol Appl. 2019 Oct 31;13(2):388–99. doi: 10.1111/eva.12876 (PMC6976953; doi:10.1111/eva.12876)
Supplement: Supplementary file 1 [file EVA-13-388-s001.pdf]

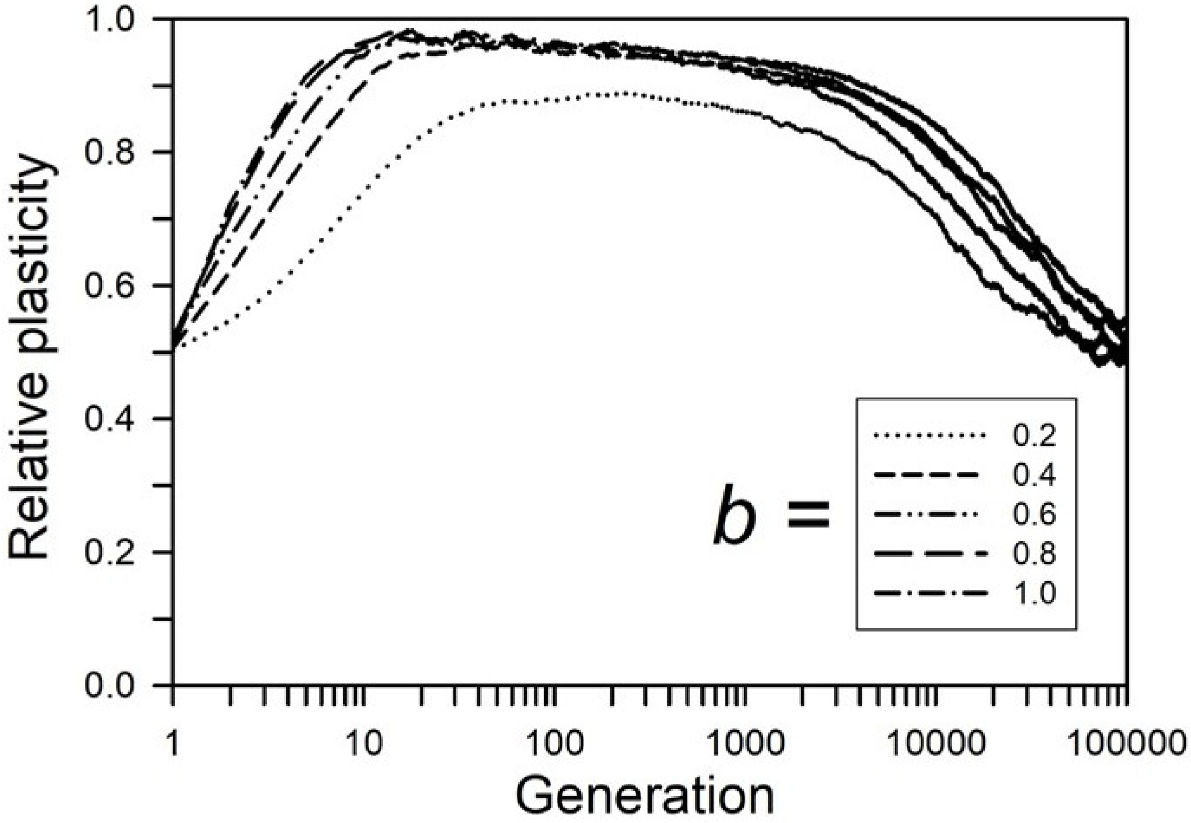

Figure S1. Temporal dynamics of the relative plasticity following a step change in the environment for different plasticity parameters (*b*), without plasticity costs and with a step change in the environment of 4.0 units. Other parameters were: $\rho = 0.5$, $\tau = 0.5$.

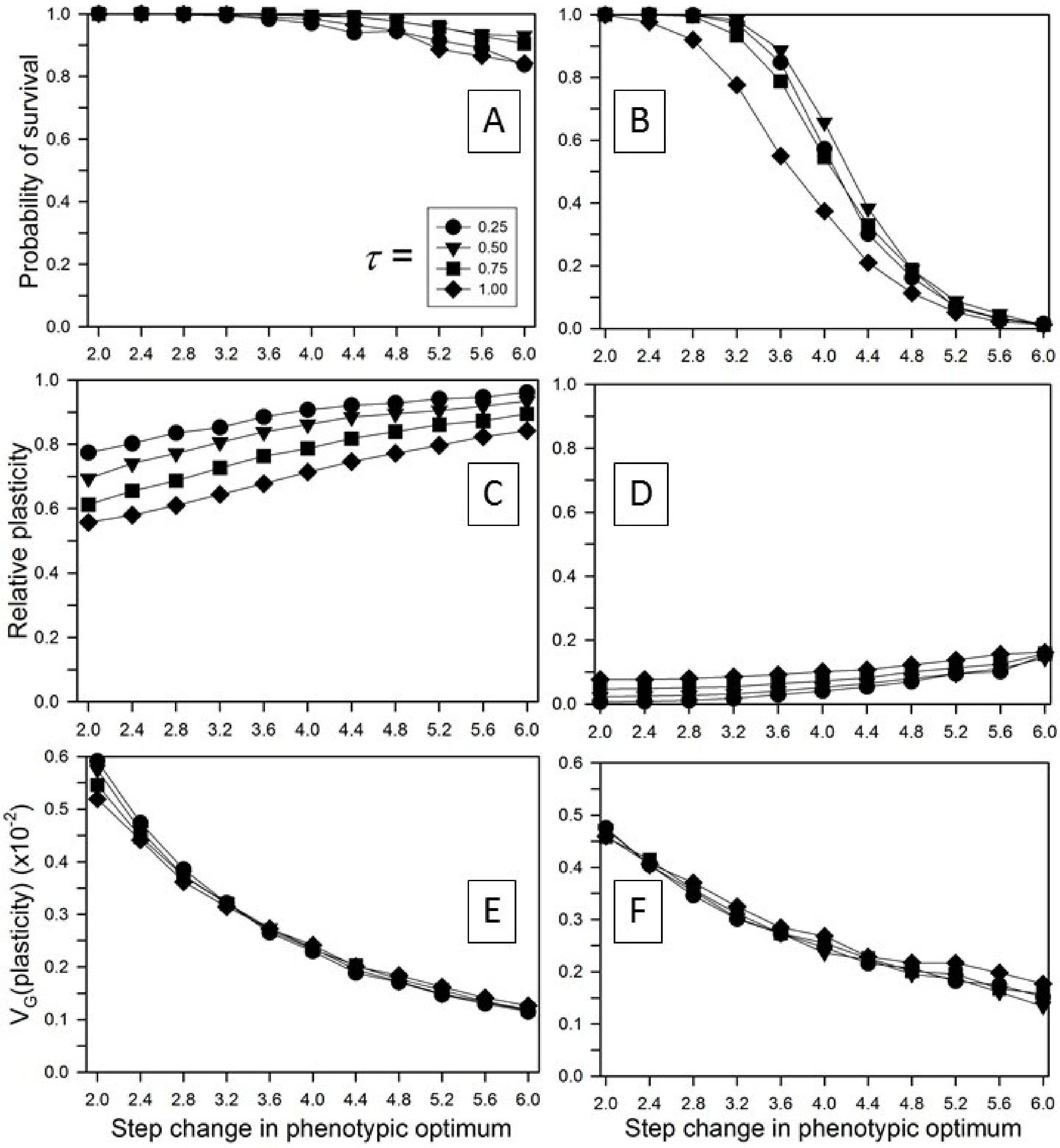

Figure S2. Responses after 1000 generations to a step change in the environment for different amounts of within-generation environmental variation (τ) before and after the step change. Other parameters were: $\rho = 0.5$. (A, C, E) Without plasticity costs and with a plasticity of $b = 0.2$. (B, D, F) With plasticity costs and a plasticity of $b = 0.6$. (A, B) The probability of survival. (C, D) Final relative plasticity. (E, F) Final genetic variation for plasticity ($\sum P_{ijk}$).

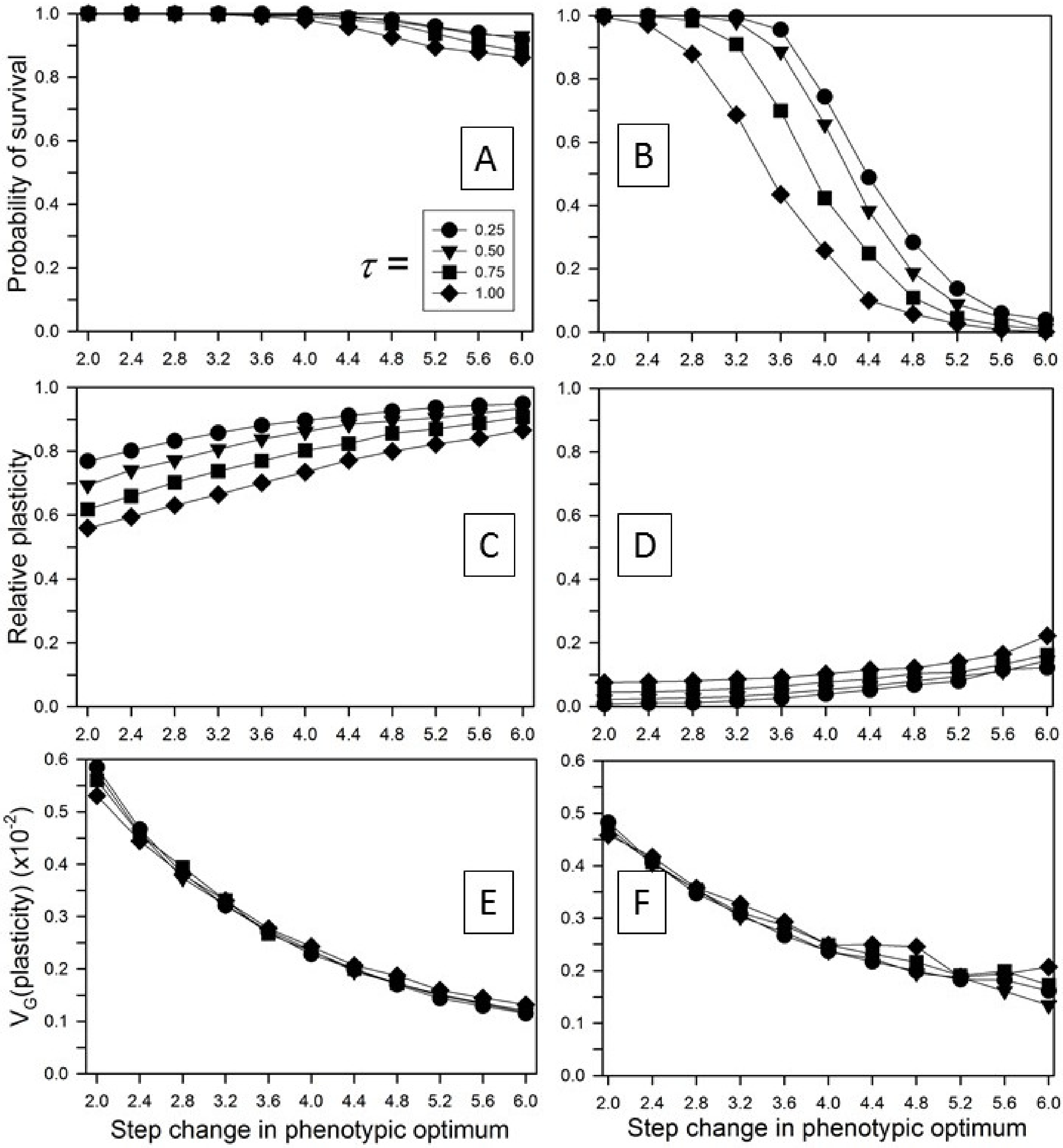

Figure S3. Responses after 1000 generations to a step change in the environment for different amounts of within-generation environmental variation (τ) following the step change. The environmental variation before the step change was 0.5. Other parameters were: $\rho = 0.5$. (A, C, E) Without plasticity costs and a plasticity of $b = 0.2$. (B, D, F) With plasticity costs and a plasticity of $b = 0.6$. (A, B) The probability of survival. (C, D) Final relative plasticity. (E, F) Final genetic variation for plasticity ($\sum P_{ijk}$).

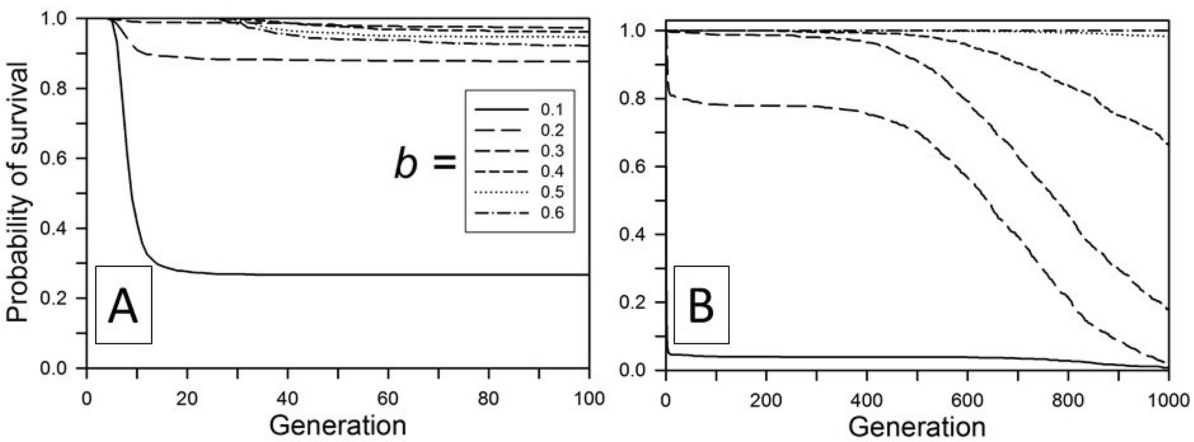

Figure S4. For continual environmental change, the probability of survival for different plasticity parameters (*b*) as a function of the time since the start of the change: (A) without plasticity costs and a rate of environmental change of 1.2 units/generation; (B) with plasticity costs and a rate of environmental change of 0.06 units/generation. Other parameters were: $\rho = 0.5$, $\tau = 0.5$.

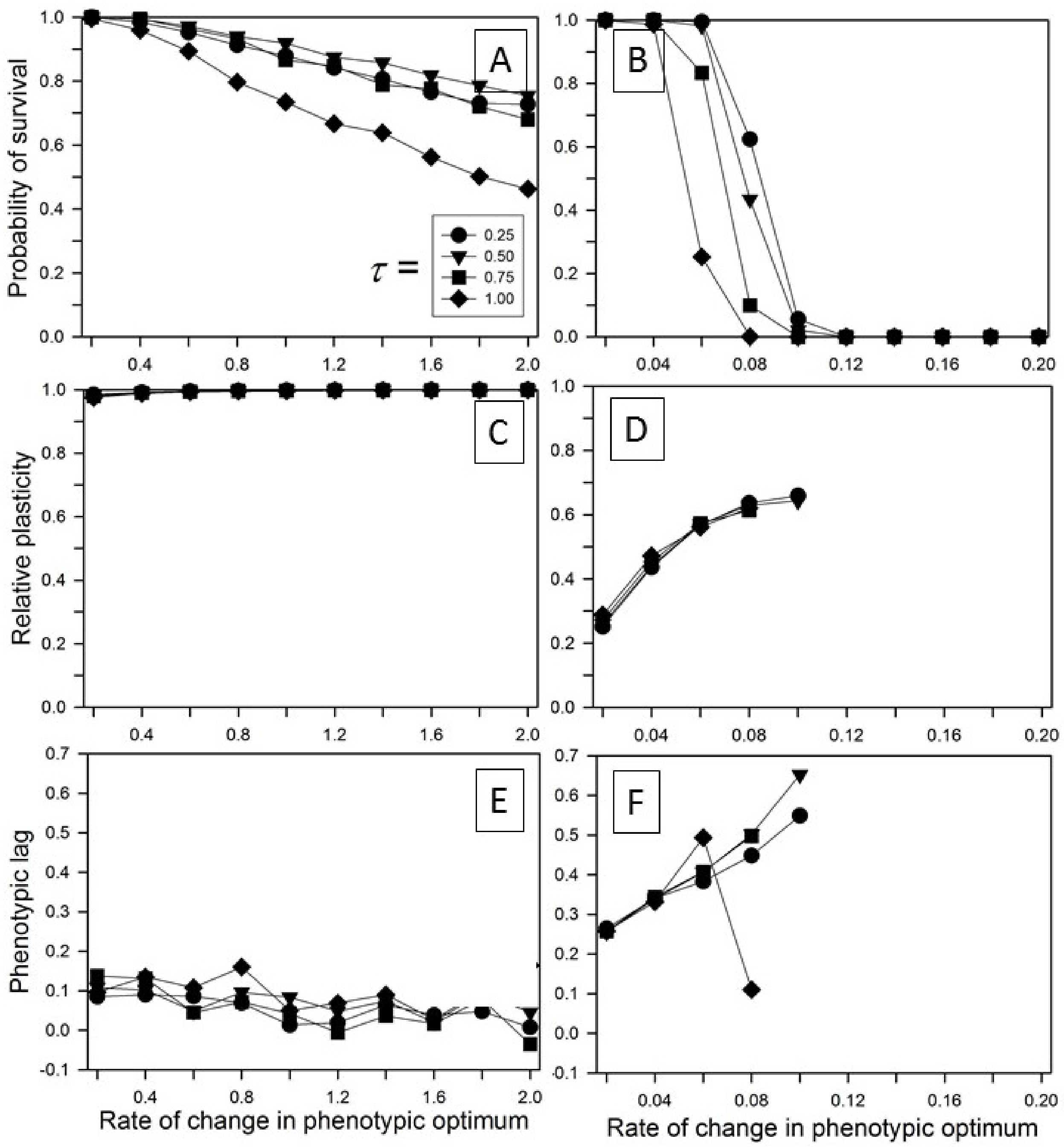

Figure S5. Responses after 1000 generations of continual environmental change for different amounts of within-generation environmental variation (τ) before and after the step change. Other parameters were: $\rho = 0.5$. (A, C, E) Without plasticity costs and $b = 0.2$. (B, D, F) With plasticity costs and $b = 0.5$. (A, B) The probability of survival. (C, D) Average final relative plasticity. (E, F) Average final phenotypic lag (expected values of optimum phenotype minus average phenotype).

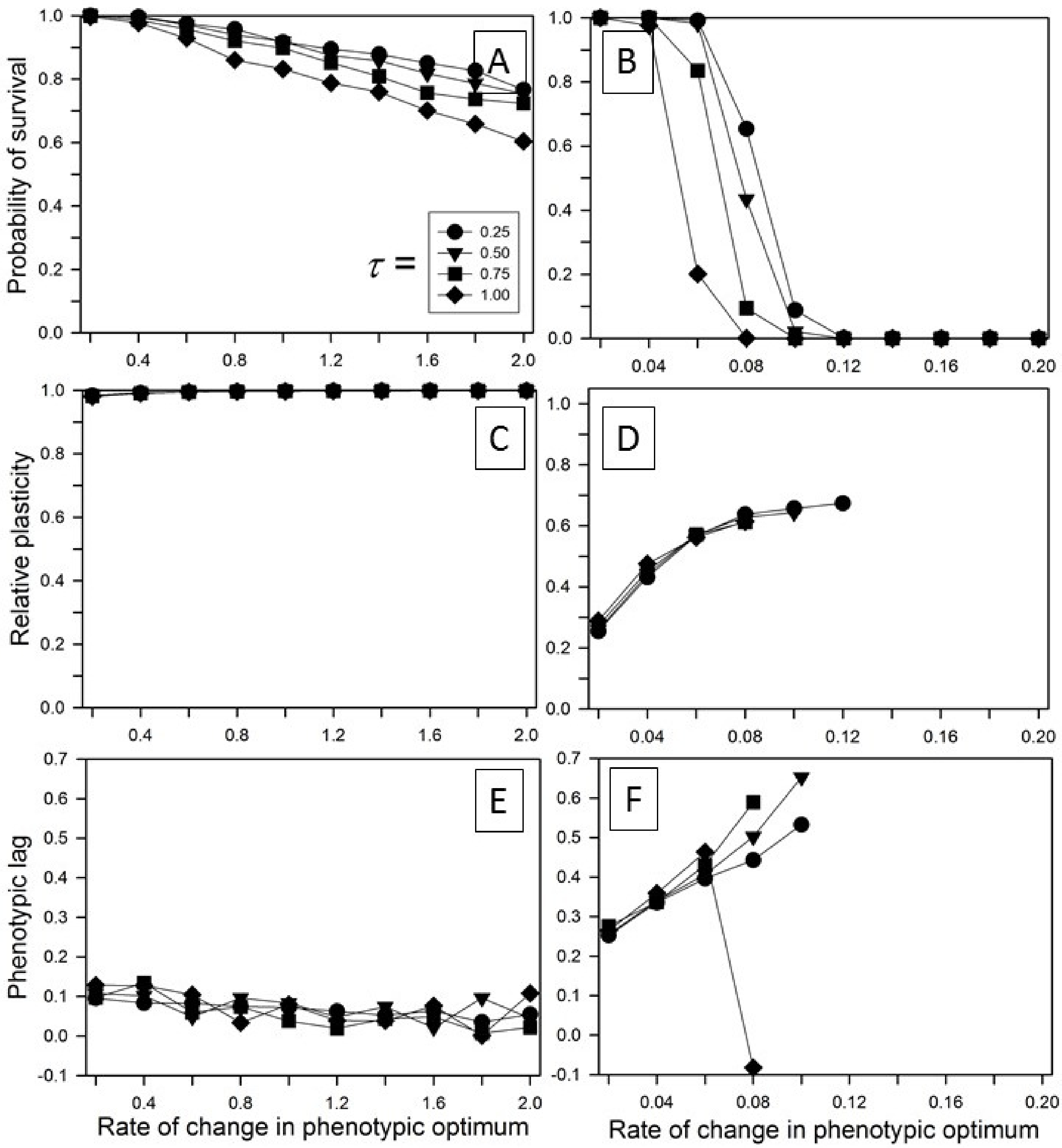

Figure S6. Responses after 1000 generations of continual environmental change for different amounts of within-generation environmental variation (τ) following the step change. The environmental variation before the step change was 0.5. Other parameters were: $\rho = 0.5$. (A, C, E) Without plasticity costs and $b = 0.2$. (B, D, F) With plasticity costs and $b = 0.5$. (A, B) The probability of survival. (C, D) Average final relative plasticity. (E, F) Average final phenotypic lag (average optimum phenotype minus average phenotype).
